# Cardiovascular Risk Assessment in Angolan Adults: A Descriptive Analysis from CardioBengo, a Community-Based Survey

**DOI:** 10.1155/2018/2532345

**Published:** 2018-09-06

**Authors:** João M. Pedro, Miguel Brito, Henrique Barros

**Affiliations:** ^1^Centro de Investigação em Saúde de Angola (CISA), Caxito, Angola; ^2^EPIUnit, Instituto de Saúde Pública, Universidade do Porto, Rua das Taipas, No. 135, 4050-600 Porto, Portugal; ^3^Health and Technology Research Center, Escola Superior de Tecnologia da Saúde de Lisboa, Instituto Politécnico de Lisboa, Av. D. João II, Lote 4.69.01, 1990-096 Lisboa, Portugal; ^4^Faculdade de Medicina, Universidade do Porto, Al. Prof. Hernâni Monteiro, 4200-319 Porto, Portugal

## Abstract

From a community-based survey conducted in Angola, 468 individuals aged 40 to 64 years and not using drug therapy were evaluated according to the World Health Organisation STEPwise Approach to Chronic Disease Risk Factor Surveillance. Using data from tobacco use, blood pressure, blood glucose, and total cholesterol levels, we estimated the 10-year risk of a fatal or nonfatal major cardiovascular event and computed the proportion of untreated participants eligible for pharmacological treatment according to clinical values alone and total cardiovascular risk. The large majority of participants were classified as having a low (<10%) 10-year cardiovascular risk (87.6%), with only 4.5% having a high (≥ 20%) cardiovascular risk. If we consider the single criteria for hypertension, 48.7% of the population should be considered for treatment. This value decreases to 22.0% if we apply the risk prediction chart. The use of hypoglycaemic drugs does not present any differences (19.0% in both situations). The use of lipid-lowering drugs (3.8%) is only recommended by the risk prediction chart. This study reveals the need of integrated approaches for the treatment of cardiovascular disorders in this population. Risk prediction charts can be used as a way to promote a better use of limited resources.

## 1. Introduction

Cardiovascular diseases (CVD) caused 17.9 million deaths worldwide in 2015, a number that has increased globally by 12.5% since 2005, with almost 80% of these deaths occurring in low and middle-income countries [1]. Their common occurrence and associated mortality, loss of independence and productivity, impaired quality of life, and social and economic costs are compelling reasons for public health concern globally [2].

The epidemiology of CVD is distinctly different in sub-Saharan Africa (SSA), compared to the rest of the world, where an unprecedented decline in mortality and a corresponding increase in the life expectancy at birth are shifting the epidemiological landscape of the region [3]. Countries in this region, where Angola is located, face a double burden as they struggle to cope with noncommunicable and infectious diseases associated with lack of socioeconomic development [3].

In 1990 the percentage of deaths by CVD in the region was 7.5% (the fourth cause of death), rising to 11.2% in 2015, being the third cause of mortality [4]. Myocardial infarctions, together with strokes, were responsible for 10.8% of deaths among females and 8.6% in males, in 2015 in SSA [4]. This represents an increase in mortality of 38.0% for stroke and 52.1% for myocardial infarction in both sexes, since 1990 [4], revealing a rising trend of CVD in SSA. The estimates made for Angola follow the same pattern, with strokes being responsible for 5.4% and myocardial infarction for 4.7% of all deaths, in 2015, the fourth and sixth cause of mortality in Angola, respectively [4].

Risk factors for CVD are shared with the majority of noncommunicable diseases and are due to exposure to behavioural risk factors: tobacco and alcohol consumption, unhealthy diet, and physical inactivity, individually modifiable. These unhealthy behaviours influence metabolic pathways and ultimately result in intermediate risk factors: obesity, hypertension, diabetes, and dyslipidaemia [5–7].

Common approaches that address behavioural and metabolic risk factors, which often coexist in the same person and act synergistically to increase an individual's total risk, are proven effective for prevention [6, 7], but in some settings, the risk factors are tackled one-by-one, without a common strategy. To design the right strategy for an individual, it is important to realise his/her “global risk” of developing CVD [7–9]. The World Health Organization (WHO) and the International Society of Hypertension (ISH) created a risk prediction chart [8], which can be applied in different populations.

In this short report, we aimed to quantify the proportion of individuals eligible for pharmacological treatment for hypertension, diabetes, and hypercholesterolemia according to single risk factor and total cardiovascular risk approaches, according to the WHO-ISH risk prediction chart.

## 2. Materials and Methods

The present report is a subset analysis from CardioBengo, a community-based study conducted in the catchment area of the Dande-Health Demographic Surveillance System (Dande-HDSS), located in the Dande Municipality, in Bengo Province, Angola [10]. This survey, done between September 2013 and March 2014, constitutes a larger baseline on cardiovascular risk factors [11], using the methodology proposed by the WHO STEPwise approach to Surveillance (STEPS) to Chronic Disease Risk Factor manual (core and expanded version 3.0) [12].

### 2.1. Sample Characterization

The subset of individuals from the CardioBengo study considered for this analysis follows the WHO-ISH risk prediction chart criteria of application [8]. The initial CardioBengo sample was made by 2,484 individuals (15 to 64 years old), drawn as a representative sex- and age-stratified random sample from the Dande-HDSS population database as previously described [11, 13].

The WHO-ISH risk prediction chart criteria of application [8] only consider individuals aged 40 or older. In the initial set, only 688 participants are aged above 40 years. From this group, 123 were excluded due to missing information in any of the parameters considered in the WHO-ISH risk prediction chart (current smoking status, systolic blood pressure, total blood cholesterol, and diabetes), and another 97 were excluded because they were already receiving pharmacological treatment for any of the three conditions (hypertension, diabetes, and hypercholesterolaemia). In this way, the final sample consists of 468 participants.

### 2.2. Data Collection

Participants were evaluated by trained interviewers and certified health professionals. As previously described in the CardioBengo study protocol [11], information on age and tobacco consumption was collected through an interview, and blood pressure and all clinical measurements (measured only in participants with overnight fasting) were obtained with the use of point of care devices, namely, automatic sphygmomanometer OMRON M6 Comfort (OMRON Healthcare Europe BV, Hoofddorp, Netherlands); blood glucose meter ACCU-CHEK Aviva (Roche Diagnostic, Indianapolis, USA); and ACCUTREND Plus (Roche Diagnostic, Indianapolis, USA) with ACCUTREND CHOLESTEROL reactive strips (Roche Diagnostic, Indianapolis, USA) [11].

### 2.3. Prediction Methods and Eligibility for Pharmacological Treatment

The WHO-ISH prediction chart for the Africa D region was used to classify each participant regarding the individual absolute cardiovascular risk [8], based on sex (male or female), age (40–49, 50–59 and ≥ 60 years), current smoking status (nonsmoker or smoker), systolic blood pressure (SBP) (120-139, 140–159, 160–179, and ≥ 180 mmHg), total blood cholesterol (4, 5, 6, 7, and 8 mmol/l), and diabetes (presence or absence, considering the WHO cut point of 6.9 mmol/l [14]).

The WHO-ISH prediction charts estimate the 10-year risk of a fatal or nonfatal major cardiovascular event (myocardial infarction or stroke) expressed in five categories—≤10% (low), 10–19% (moderate), 20–29% (high), and ≥30% (very high)—in people who do not have established cardiovascular diseases [8].

The eligibility for treatment with antihypertensive, hypoglycaemic, or lipid-lowering drugs was defined according to the WHO guidelines for assessment and management of cardiovascular risk [8]. Also, eligibility for treatment according to the single risk factor approach was considered: for antihypertensive drug use SBP of ≥140 mmHg [15, 16]; for hypoglycaemic drug use fasting blood glucose >6.9 mmol/l [14]; and for lipid-lowering drugs use a total blood cholesterol level ≥8 mmol/l [9].

### 2.4. Statistical Analysis

Data were double entered into a PostgreSQL® database and imported into SPSS® version 23 (IBM, New York, USA) for data analysis. Descriptive data are reported as absolute frequencies and percentages. We estimated the proportion of participants classified in different categories of total cardiovascular risk, as well as the proportion of subjects eligible for pharmacological treatment (according to the different criteria), by sex. We consider a 95% confidence interval (95% CI) for all proportions calculated.

### 2.5. Ethical Approval

All procedures performed in this study were in accordance with the standards of the Ethics Committee of the Angolan Ministry of Health and with the 1964 Helsinki declaration and its later amendments. Written informed consent was obtained from all participants.

## 3. Results

The majority of the population in this study was female (69.7%) following the structure of the Dande-HDSS [10], with 17.9% of the population aged above 60 years. Smoking was more frequent among men (18.3% versus 7.4% in women) with almost half the population (48.7%) presenting a SBP ≥140 mmHg, with a higher occurrence in women (52.5% versus 40.1% in men). None of the individuals presented total blood cholesterol >8 mmol/l, with only women presenting values >7 mmol/l (6.1%). The prevalence of diabetes was similar among men and women (18.4% in women and 20.4% in men), with almost 10% of the population having both SBP ≥140 mmHg and diabetes (Table 1).

Most of the participants (87.6%) were classified as having low (<10%) 10-year cardiovascular risk. The frequencies were 7.9%, 2.6%, and 1.9% for the cardiovascular risk categories 10–19 (moderate), 20–29 (high), and ≥30% (very high), respectively. Women presented higher frequencies than men in all cardiovascular risk categories above the moderate level and the total cardiovascular risk increased with age (Table 2).

Considering only the criteria of SBP ≥140 mmHg, 48.7% of the population should be considered for treatment with antihypertensive drugs, but if we apply the WHO/ISH criteria based on the prediction chart, this number will decrease by more them half to 22.0%. On the other hand, the use of lipid-lowering drugs is not considered for any individual by the singular criteria, but if we apply the prediction chart, this number rises to 3.8% (Table 3).

## 4. Discussion

Only 4.5% of the population was classified as having at least a high (≥20%) cardiovascular risk. These results are in accordance with those described in other populations of the continental sub-Saharan Africa, namely, 3.7% in Mozambique [17] and 5% in Nigeria [18], or in other low and middle-income countries in Asia (4.9% in rural India, 1.3% in Cambodia, 2.3% in Malaysia, and 6% in Mongolia) [19, 20]. In all these surveys, the accumulation of risk factors exists in different levels. In this analysis, almost one-tenth of the population accumulates at least two major risk factors (hypertension and diabetes).

This raises additional concerns when we analyse this subset of results together with the values found in the CardioBengo population aged 16 to 64 years: a prevalence of hypertension of 18.0%, diabetes of 9.2%, and hypercholesterolaemia of 4.0%, with a low awareness and control levels [13]. The CVD are present in this population, but this does not imply that the health systems in the region are ready to face with this growing reality.

According to the WHO guidelines for assessment and management of cardiovascular diseases [8], at least one-quarter of the participants in this report would be eligible for treatment with antihypertensive drugs, whereas if only the single criteria were applied for hypertension, almost half would be considered for pharmacological treatment. Even if some discussion is possible about the applicability of pharmacological treatment with a SBP of 140, the specific behaviour of blood pressure in black individuals (as the population observed) should be considered [16].

The use of the WHO-ISH risk prediction chart has already been proven as more cost effective than other approaches that make treatment decisions based on individual risk factor thresholds only, especially for hypertension management [7, 21–23]. In a South-African study, different strategies for initiation of drug treatment were tested, and the conclusion points to the fact that hypertension treatment based on the total cardiovascular risk is more effective at saving lives and less costly than those based only on the BP level [24].

In this prediction chart, we included the total cholesterol measurement; however, the WHO-ISH chart offers the possibility of not using this measurement [8]. The use of laboratory (or point of care) assessment in low-resource settings is dispensable, avoiding additional costs to the system without losing a significant predictive power or introducing an overconsumption of drugs, allowing for a better targeting of resources to those who are more likely to develop CVD [25, 26].

The high percentage of individuals that requires pharmacological treatment for diabetes (19.0%, very similar between gender) is alarming, considering the fact that diabetes alone is a very important health condition, but also an important risk factor for CVD; adults with diabetes have a two or three times higher rate of CVD than adults without diabetes [8, 27].

Health promotion initiatives such as smoking cessation and individual advisement on specific lifestyle and diet control are also strategies necessary to align with the use of pharmacological treatments. Even if some of the proposed methodologies (statins for hypercholesterolemia, for example) cannot be easily applied due to the steady supply of drugs, changes to personal lifestyle should be encouraged, with good results in the decrease of the global cardiovascular risk [18, 21, 22].

The focus of targeting high-risk people through a total cardiovascular risk approach instead of a single risk factor approach reduces health care expenditure by reducing drug costs [18]. However, to diminish CVD burden in the entire population in a sustainable way, wider interventions are needed. The focus should be on the primary healthcare, where the majority of the individuals have access, allowing for the promotion of opportunistic screening of CVD risk factors and patient registration and tracking, with a better use of available resources [23].

The findings in this report should be interpreted cautiously. The study was conducted only at a district-level, and due to the criteria of application of the WHO-ISH risk prediction chart the sample size was small. Even with these limitations, this report adds evidence to the emerging public health issue of CVD, especially in older individuals, and provides additional information on the impact of the use of risk prediction charts.

## 5. Conclusion

The use of the WHO-ISH guidelines for cardiovascular risk prediction reduces the population eligible for pharmacological treatment, giving prominence to an integrated approach based on lifestyle changes. This may sound appropriate to a low-resource setting, but the low education level of this population and the lower ability in older individuals for altering behaviours are factors to consider in future interventions. The training of health professionals and raising awareness may be the starting point to tackle CVD in this population, where the use of these charts can have a role to play in the wise use of resources available.

## Figures and Tables

**Table 1:** Characteristics of the participants and prevalence by sex.

	**Total (*n *= 468)**	**Female (*n *= 326)**	**Male (*n *= 142)**
	%** (95% CI)**	%** (95% CI)**	%** (95% CI)**
**Age (years)**			
40-49	36.3 (32.1-40.8)	35.0 (30.0-40.3)	39.4 (31.7-47.6)
50-59	45.7 (41.2-50.2)	47.9 (42.5-53.3)	40.8 (33.1-49.0)
≥ 60	17.9 (14.7-21.6)	17.2 (13.5-21.7)	19.7 (14.0-27.0)
**Current smoking status**			
Non-smoker	89.3 (86.2-91.8)	92.6 (89.2-95.0)	81.7 (74.5-87.2)
Smoker	10.7 (8.2-13.8)	7.4 (5.0-10.8)	18.3 (12.8-25.5)
**Systolic blood pressure (mmHg)**			
120-139	51.3 (46.8-55.8)	47.5 (42.1-52.9)	59.9 (51.7-67.6)
140-159	26.7 (22.9-30.9)	26.7 (22.2-31.8)	26.8 (20.2-34.6)
160-179	14.3 (11.4-17.8)	16.6 (13.0-21.0)	9.2 (5.5-15.1)
≥ 180	7.7 (5.6-10.4)	9.2 (6.5-12.8)	4.2 (1.9-8.9)
**Total blood cholesterol (mmol/l)**			
4	55.6 (51.1-60.0)	52.1 (46.7-57.5)	63.4 (55.2-70.9)
5	27.1 (23.3-31.3)	26.7 (22.2-31.8)	28.2 (21.4-36.1)
6	13.0 (10.3-16.4)	15.0 (11.5-19.3)	8.5 (4.9-14.2)
7	4.3 (2.8-2.8)	6.1 (4.0-9.2)	0
8	0	0	0
**Diabetes** ^a^			
Absence	81.0 (77.2-84.3)	81.6 (77.0-85.4)	79.6 (72.2-85.4)
Presence	19.0 (15.7-22.8)	18.4 (14.6-23.0)	20.4 (14.6-27.8)
**With SBP >140 mmHg and Diabetes**	9.8 (7.4-12.8)	9.5 (6.8-13.2)	10.6 (6.5-16.7)

^a^Fasting blood glucose ≥6.9 mmol/l.

**Table 2:** Distribution of 10-year risk of a fatal or nonfatal major cardiovascular event, according to sex and age.

	**Total, **%** (95% CI)**			
	**≤10**%	**10-19**%	**20-29 **%	**≥30**%
	**Low**	**Moderate **	**High**	**Very High**
**40-49 years**	34.2 (30.0-38.6)	1.3 (0.6-2.8)	0.6 (0.2-1.9)	0.2 (0.0-1.2)
**50-59 years**	40.6 (36.2-45.1)	3.6 (2.3-5.7)	0.6 (0.2-1.9)	0.9 (0.3-2.2)
**≥ 60 years**	12.8 (10.1-16.2)	3.0 (1.8-5.0)	1.3 (0.6-2.8)	0.9 (0.3-2.2)
**Total**	**87.6 (84.3-90.3)**	**7.9 (5.8-10.7)**	**2.6 (1.5-4.4)**	**1.9 (1.0-3.6)**

	**Female, **%** (95% CI)**			
	**≤10**%	**10-19**%	**20-29 **%	**≥30**%
	**Low**	**Moderate **	**High**	**Very High**

**40-49 years**	32.5 (27.7-37.8)	1.5 (0.7-3.5)	0.6 (0.2-2.2)	0.3 (0.1-1.7)
**50-59 years**	41.7 (36.5-47.1)	4.0 (2.3-6.7)	0.9 (0.3-2.7)	1.2 (0.5-3.1)
**≥ 60 years**	11.7 (8.6-15.6)	3.1 (1.7-5.6)	1.8 (0.8-4.0)	0.6 (0.2-2.2)
**Total**	**85.9 (81.7-89.3)**	**8.6 (6.0-12.1)**	**3.4 (1.9-5.9)**	**2.1 (1.0-4.4)**

	**Male, **%** (95% CI)**			
	**≤10**%	**10-19**%	**20-29 **%	**≥30**%
	**Low**	**Moderate **	**High**	**Very High**

**40-49 years**	38.0 (30.5-46.2)	0.7 (0.1-3.9)	0.7 (0.1-3.9)	-^a^
**50-59 years**	38.0 (30.5-46.2)	2.8 (1.1-7.0)	-^a^	-^a^
**≥ 60 years**	15.5 (10.5-22.3)	2.8 (1.1-7.0)	-^a^	1.4 (0.4-5.0)
**Total**	**91.5 (85.8-95.1)**	**6.3 (3.4-11.6)**	**0.7 (0.1-3.9)**	**1.4 (0.4-5.0)**

^a^No individuals in this category.

**Table 3:** Frequencies of individuals who require pharmacological treatment by sex.

	**Single risk criteria**			**Considering WHO/IS** **H** ^**a**^ ** risk prediction**		
	**Total, **%	**Female, **%	**Male, **%	**Total, **%	**Female, **%	**Male, **%
	**(95**%** CI)**	**(95**%** CI)**	**(95**%** CI)**	**(95**%** CI)**	**(95**%** CI)**	**(95**%** CI)**
**Antihypertensive ** **drugs**	48.7	52.5	40.1	22.0	25.8	13.4
	(44.2-53.2)	(47.0-57.8)	(32.4-48.4)	(18.5-26.0)	(21.3-30.8)	(8.7-20.0)
**Lipid-lowering ** **drugs**	-^b^	-^b^	-^b^	3.8	4.9	1.4
				(2.4-6.0)	(3.0-7.8)	(0.4-5.0)
**Hypoglycaemic ** **drugs**	19.0	18.4	20.4	19.0	18.4	20.4
	(15.7-22.8)	(14.6-23.0)	(14.6-27.8)	(15.7-22.8)	(14.6-23.0)	(14.6-27.8)

^a^World Health Organization/International Society of Hypertension.

^b^No individuals in this category.

## Data Availability

The data used to support the findings of this study are available from the corresponding author upon request.

## References

[1] Wang H., Naghavi M., Allen C. (2016). Global, regional, and national life expectancy, all-cause mortality, and cause-specific mortality for 249 causes of death, 1980–2015: a systematic analysis for the Global Burden of Disease Study 2015. *Lancet*.

[2] Labarthe D. (2011). *Epidemiology And Prevention of Cardiovascular Diseases: A Global Challenge*.

[3] Jamison D. T., Feachem R. G., Makgoba M. W. (2006). *Disease and Mortality in Sub-Saharan Africa*.

[4] Institute for Health Metrics and Evaluation. GBD compare - Viz Hub, 2018, http://vizhub.healthdata.org/gbd-compare/

[5] World Health Organization *Global status report on Noncommunicable diseases. Geneva: World Health Organization*.

[6] World Health Organization (2011). *Global Atlas on Cardiovascular Disease Prevention And Control*.

[7] World Health Organization *Global health risks: Mortality and burden of disease attributable to selected major risks. Geneva: World Health Organization*.

[8] World Health Organization *Prevention of Cardiovascular Disease: Guidelines for Assessment And Management of Cardiovascular Risk. Geneva: World Health Organization*.

[9] World Health Organization *Global Action Plan for The Prevention And Control of Noncommunicable Diseases 2013–2020. Geneva: World Health Organization*.

[10] Costa M. J., Rosário E., Langa A., António G., Bendriss A., Nery S. V. (2012). Setting up a demographic surveillance system in Northern Angola. *Etude de la Population Africaine*.

[11] Pedro J. M., Rosário E., Brito M., Barros H. (2016). CardioBengo study protocol: A population based cardiovascular longitudinal study in Bengo Province, Angola. *BMC Public Health*.

[12] World Health Organization *The STEPS Instrument and Support Materials*.

[13] Pedro J. M., Brito M., Barros H. (2018). Prevalence, awareness, treatment and control of hypertension, diabetes and hypercholesterolaemia among adults in Dande municipality, Angola. *Cardiovascular Journal of Africa*.

[14] World Health Organization (2006). *Definition and diagnosis of diabetes mellitus and intermediate hyperglycaemia: report of a WHO/IDF consultation*.

[15] Weber M. A., Schiffrin E. L., White W. B. (2014). Clinical Practice Guidelines for the Management of Hypertension in the Community. *The Journal of Clinical Hypertension*.

[16] Flack J. M., Sica D. A., Bakris G. (2010). Management of high blood pressure in Blacks: an update of the International Society on Hypertension in Blacks consensus statement. *Hypertension*.

[17] Damasceno A., Padrão P., Silva-Matos C., Prista A., Azevedo A., Lunet N. (2013). Cardiovascular risk in Mozambique: Who should be treated for hypertension?. *Journal of Hypertension*.

[18] Mendis S., Lindholm L. H., Anderson S. G. (2011). Total cardiovascular risk approach to improve efficiency of cardiovascular prevention in resource constrain settings. *Journal of Clinical Epidemiology*.

[19] Ghorpade A. G., Shrivastava S. R., Kar S. S., Sarkar S., Majgi S. M., Roy G. (2015). Estimation of the cardiovascular risk using world health organization/international society of hypertension (WHO/ISH) risk prediction charts in a rural population of South India. *International Journal of Health Policy and Management*.

[20] Otgontuya D., Oum S., Buckley B. S., Bonita R. (2013). Assessment of total cardiovascular risk using WHO/ISH risk prediction charts in three low and middle income countries in Asia. *BMC Public Health*.

[21] World Health Organization *Prevention and control of noncommunicable diseases: guidelines for primary health care in low-resource settings. Geneva: World Health Organization*.

[22] Modesti P. A., Agostoni P., Agyemang C. (2014). Cardiovascular risk assessment in low-resource settings: a consensus document of the European Society of Hypertension Working Group on Hypertension and Cardiovascular Risk in Low Resource Settings. *Journal of Hypertension*.

[23] Bovet P., Chiolero A., Paccaud F., Banatvala N. (2015). Screening for cardiovascular disease risk and subsequent management in low and middle income countries: Challenges and opportunities. *Public Health Reviews*.

[24] Gaziano T. A., Steyn K., Cohen D. J., Weinstein M. C., Opie L. H. (2005). Cost-effectiveness analysis of hypertension guidelines in South Africa: Absolute risk versus blood pressure level. *Circulation*.

[25] Pandya A., Weinstein M. C., Salomon J. A., Cutler D., Gaziano T. A. (2014). Who needs laboratories and who needs statins? Comparative and cost-effectiveness analyses of non-laboratory-based, laboratory-based, and staged primary cardiovascular disease screening guidelines. *Circulation: Cardiovascular Quality and Outcomes*.

[26] Nordet P. R., Mendis S., Dueñas A. (2013). Total cardiovascular risk assessment and management using two prediction tools, with and without blood cholesterol. *MEDICC Review*.

[27] *Global Report on Diabetes. Geneva: World Health Organization*.

